# The DnaA Tale

**DOI:** 10.3389/fmicb.2018.00319

**Published:** 2018-02-28

**Authors:** Flemming G. Hansen, Tove Atlung

**Affiliations:** ^1^Department of Bioengineering, Technical University of Denmark, Lyngby, Denmark; ^2^Department of Science and Environment, Roskilde University, Roskilde, Denmark

**Keywords:** DnaA protein, DnaA box, *dnaA* gene, DnaA ADP/ATP, *oriC*, initiation control models, initiator titration, cell cycle

## Abstract

More than 50 years have passed since the presentation of the Replicon Model which states that a positively acting initiator interacts with a specific site on a circular chromosome molecule to initiate DNA replication. Since then, the origin of chromosome replication, *oriC*, has been determined as a specific region that carries sequences required for binding of positively acting initiator proteins, DnaA-boxes and DnaA proteins, respectively. In this review we will give a historical overview of significant findings which have led to the very detailed knowledge we now possess about the initiation process in bacteria using *Escherichia coli* as the model organism, but emphasizing that virtually all bacteria have DnaA proteins that interacts with DnaA boxes to initiate chromosome replication. We will discuss the *dnaA* gene regulation, the special features of the *dnaA* gene expression, promoter strength, and translation efficiency, as well as, the DnaA protein, its concentration, its binding to DnaA-boxes, and its binding of ATP or ADP. Furthermore, we will discuss the different models for regulation of initiation which have been proposed over the years, with particular emphasis on the Initiator Titration Model.

## Introduction

**Figure 1:**
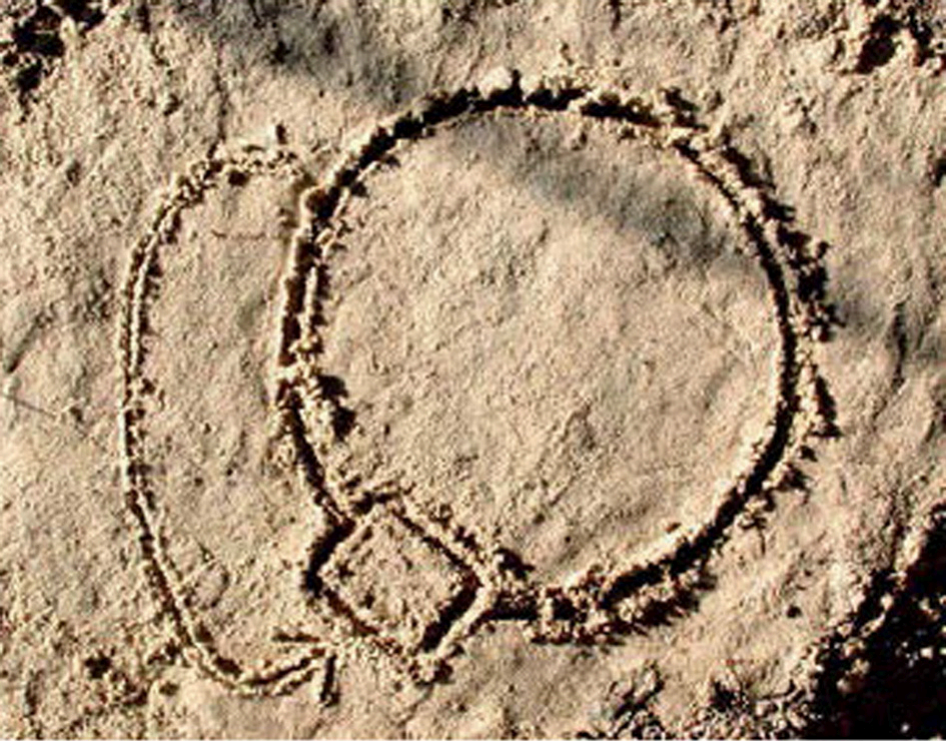
The replicon model. A drawing in the sand (provided by M. Mechali to an EMBO meeting report by Skarstad et al. (2003, Copyright, John Wiley and Sons) to celebrate the 40th anniversary of the model.

The Replicon Model hypothesizes that to initiate DNA replication a positively acting initiator molecule interacts with a specific site, the replicator, on a circular chromosome (Figure 1; Jacob et al., 1963). The model was probably designed based on two main findings: (1) that the bacterial chromosome was circular (Cairns, 1963); and (2) that arrest of protein and/or RNA synthesis led to inhibition of initiation but allowed termination of ongoing rounds of replication (Maaløe and Hanawalt, 1961).

Since then, several models, to be addressed later, have been designed to explain some of the characteristics of the coupling between the cell cycle and the growth of *Escherichia coli* and possibly also several other bacteria. *Escherichia coli* can grow at very different growth rates depending on the carbon source and other nutrients present in the growth medium. The generation time in rich media, such as LB medium, is ~20 min at 37°C whereas generation times in minimal media with poor carbon sources can be several hours. At all these generation times it is characteristic for *E. coli* (and most other bacteria) that fast growing cells are big and slowly growing cells are small, but the size of a baby cell will always be very close to half the size of the mother cell independent of the growth rate (Schaechter et al., 1958; Maaløe and Kjeldgaard, 1966).

It should be emphasized that the present review is the story about DnaA and *oriC*. For more detailed reviews see e.g., Messer (2002), Skarstad and Katayama (2013), or Leonard and Grimwade (2015).

## The cell cycle

A very important early input to characterization of the bacterial cell cycle was obtained in the work with the baby cell machine; a membrane elution technique (Helmstetter and Cummings, 1963, 1964). In this method the cells of an exponentially growing culture are attached to a membrane filter, the filter holder is inverted and growth medium is passed through the filter. The bacteria grow on the filter and one daughter cell detaches upon cell division and is collected. The main findings were that if cells grew with generation times shorter than 60 min the time it took to replicate the chromosome was close to 40 min, this was termed the C-period, and that the time it took from termination of chromosome replication to cell division was close to 20 min, termed the D-period (Helmstetter, 1967; Helmstetter and Cooper, 1968; Helmstetter et al., 1968). These primary studies were carried out using the *Escherichia coli* B/r strain that behaved very well in the baby cell machine, i.e., the mother cells were firmly attached to the filter (Helmstetter and Cummings, 1963, 1964). Two different approaches have been used in baby cell experiments. One where DNA was labeled with radioactivity before the cells were attached to the filter and radioactivity was determined in the effluent at different times, the other (and conceptually simpler) where newborn cells were collected on ice to stop growth and an experiment was started with bacterial babies transferred to the desired growth temperature getting synchronized cell division (Clark and Maaløe, 1967). This has been the preferred method for later experiments addressing cell cycle parameters. Cells with generation times longer than 60 min very often have a period with only one non-replicating chromosome, this period was termed the B-period (Helmstetter and Pierucci, 1976). Cells growing with generation times longer than 60 min have longer C- and D-periods than given above, and the B-, C-, and D-period show some variation in different strains of *E. coli* (Helmstetter, 1996; Michelsen et al., 2003).

## The initiation mass

The determination of the cell cycle parameters (see above) and the very careful measurements of DNA and cell mass in a study of macromolecular concentrations at different growth rates of *Salmonella typhimurium* (Schaechter et al., 1958) led to the suggestion that bacteria like *E. coli* and to *S. typhimurium*, initiate replication when a certain mass (or volume) is present per origin of replication, the initiation mass (Donachie, 1968) or the critical volume (Pritchard et al., 1969). There has been some dispute whether the initiation mass is the same at all growth rates (Wold et al., 1994). However, it is generally accepted that the initiation mass does not vary very much in *E. coli* K-12 growing at different growth rates (Herrick et al., 1996).

## Phenomenology of control of chromosome replication

The results obtained with the baby cell machine can only be explained if all chromosomal origins in a cell are initiated once and only once per cell cycle. The length of the C and D periods for fast growing bacteria implies that these cells contain several origins and have several ongoing replication forks. That this is the case was demonstrated clearly by flow cytometry of cultures treated with rifampicin that inhibits initiation of chromosome replication but allows ongoing replication forks to terminate (Skarstad et al., 1986). To determine origins per cell at the time of drug addition it is necessary also to stop cell division (Løbner-Olesen et al., 1989) by simultaneous addition of cephalexin (Begg and Donachie, 1985). In the fast growing cells which carried more than one origin at the time of initiation (and rifampicin addition) flow cytometry showed in addition that all origins were initiated synchronously, i.e., cells had either 2^n^ or 2^(n+1)^ replicated chromosomes (Skarstad et al., 1986) which (with simultaneous cephalexin addition) will be equal to the number of origins per cell (Skarstad et al., 1986; Løbner-Olesen et al., 1989). Thus, a model for control of chromosome replication should satisfy what we know about the cell cycle and the behavior of chromosomes and origins. However, before going into the modeling mode we will give a relatively detailed description of the *dnaA* gene and its gene product, which is a positively acting initiator, and the origin of replication, which is the replicator. In Francois Jacob's laboratory, Masamichi Kohiyama isolated temperature sensitive DNA replication mutants some of which continued to replicate at a non-permissive growth temperature until they terminated replication, presumably at the terminus of replication (Jacob et al., 1963; Kohiyama et al., 1963). Some of these mutants were *dnaA* mutants. This historical tale is about the initiator, the DnaA protein and its interaction with the replicator, the origin of replication - *oriC*, and will focus on details about the *dnaA* gene encoding the DnaA protein as well as the requirements for the interaction of the DnaA protein with *oriC*.

## The *dnaA* mutants

The temperature sensitive K-12 strains CRT46 and CRT83, were the first strains carrying mutations in the *dnaA* gene, *dnaA46*, and *dnaA83*, respectively. These mutations were genetically mapped and found to be located in the vicinity of the *ilv* locus (Hirota et al., 1968). In the following years several strains carrying mutations in the *dnaA* gene (see **Figure 5** below) were isolated in different laboratories (Kuempel, 1969; Carl, 1970; Wechsler and Gross, 1971; Beyersmann et al., 1974; Sevastopoulos et al., 1977). The PC2 strain (Carl, 1970) exhibits a slow stop phenotype similar to that of *dnaA* mutants, but carries a mutation in the *dnaC* gene that encodes the loading factor for DnaB, the DNA helicase.

## Fine mapping of the *dnaA gene*

**Figure 2:**
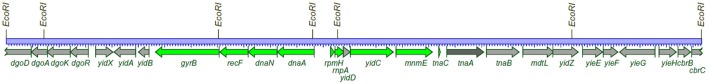
The *dnaA* region of the *E. coli* chromosome. 26,332 bp between the outmost *Eco*RI sites is the chromosomal DNA present on two λ*tna* phages (λ*tna* 552 and λ *tna* 530). The drawing is based on the published complete *E. coli* sequence version (acc.no. U00096.2). The genes (proteins) stained green are those that could be identified on SDS-polyacrylamide gels. The light gray proteins and the dark gray protein encoded by the *tnaA* gene were not visible (Hansen and von Meyenburg, 1979; Ream et al., 1980).

The genetic structure of the *dnaA* region of the chromosome was characterized using specialized transducing λ bacteriophages, λ*tna* (Hansen and von Meyenburg, 1979; Ream et al., 1980). The chromosomal genes carried on the phages were identified by recombinational rescue of mutations known to be located close to the *ilv* locus, and mapped relative to an *Eco*RI restriction enzyme map constructed from cleavage of different transducing phages. In this study the size of gene products, the proteins, were also characterized by introducing the λ*tna* phages into UV-killed bacteria which had degraded all chromosomal DNA (Murialdo and Siminovitch, 1972). The *dnaA* and *dnaN* genes were also characterized by a Japanese group (Sakakibara and Mizukami, 1980). Several recombinant plasmids were constructed using restriction enzymes and DNA from different specialized transducing λ*tna* phages. These plasmids were used to determine the precise position of more genes by complementation of mutants and characterization of gene products in the maxicell system (Sancar et al., 1979; Hansen F. G. et al., 1982; Hansen et al., 1985). The *dnaA* region as characterized by λ*tna* phages and plasmids derived from the phages is shown in Figure 2.

## The origin of replication—*oriC*

The origin of replication was found to be located close to the *ilv* genes in several studies during the 1970s (e.g., Bird et al., 1972; Marsh and Worcel, 1977). The *oriC* symbol was introduced for the origin of replication (Hiraga, 1976). A minichromosome, pSY211, carrying *oriC* was constructed by combining an ~9.5 kb *Eco*RI fragment with a non-replicating fragment conferring ampicillin resistance (Yasuda and Hirota, 1977). It was also obtained on specialized transducing λ*asn* phages (von Meyenburg et al., 1979) which could be established as minichromosomes when transduced into a recombination deficient *recA* strain lysogenic for λ and requiring asparagine. Such strains are heat-sensitive due to the presence of the λcI_857_ gene on the λ-minichromosome. Small minichromosomes, e.g., pCM959 (von Meyenburg et al., 1979; Buhk and Messer, 1983) carrying exclusively chromosomal DNA, were isolated by selecting heat-resistant Asn^+^ clones (von Meyenburg et al., 1979). Further characterization of *oriC* was carried out by deletion analysis of a plasmid carrying a fragment with a selectable marker and the 9.5 kb *Eco*RI fragment from λ*asn132* (Messer et al., 1978, 1979). In this way *oriC* could be located between a *Bam*HI and a *Xho*I site and this 422 bp region was sequenced (Meijer et al., 1979; Messer et al., 1979; Sugimoto et al., 1979)^1^. Further deletion analysis limited the *oriC* region to 245 bp (Tabata et al., 1983).

## The DnaA-boxes

**Figure 3:**
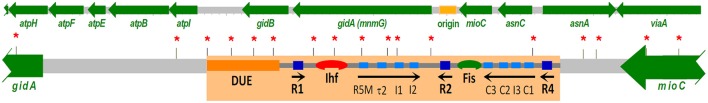
The region of the *E. coli* chromosome carrying *oriC*, the origin of replication, and neighboring genes. A recent review presents a detailed walkthrough of the various binding sites in *oriC* and the interactions with the DnaA protein and other factors in forming the orisome that is required to initiate replication (Leonard and Grimwade, 2015). DnaA boxes with high (dark blue boxes) and low (light blue boxes) affinity, respectively; positions of GATC sites (^*^). However, in an exponentially growing culture containing cells of all ages only the R1, R2, and R4 are bound by DnaA protein (Samitt et al., 1989).

The sequence of the *E. coli oriC* was the first origin of replication to be reported for a cellular organism. The next origin to be sequenced was that of *Salmonella typhimurium* (Zyskind and Smith, 1980). Similarly to the *E. coli oriC* the *Salmonella* sequence carried an unexpected high density of GATC sites (Figure 3), where the position of most were conserved when compared to *E. coli*.

Sequencing other origins of the *Enterobacteriacea* family (Zyskind et al., 1981; Cleary et al., 1982) revealed a ***r***epeated sequence (R-box) which was later renamed the DnaA-box. Since these early days hundreds of bacteria have been sequenced. Virtually all bacteria carry a *dnaA* gene. The approximate position of the origin of replication can in many cases be determined by the Base-Skew-Method (McLean et al., 1998) and in most cases the precise location of the origin can be identified by the presence of DnaA-boxes which are also present in virtually all bacteria.

## DnaA sets the initiation mass—is the master regulator of initiation

**Figure 4:**
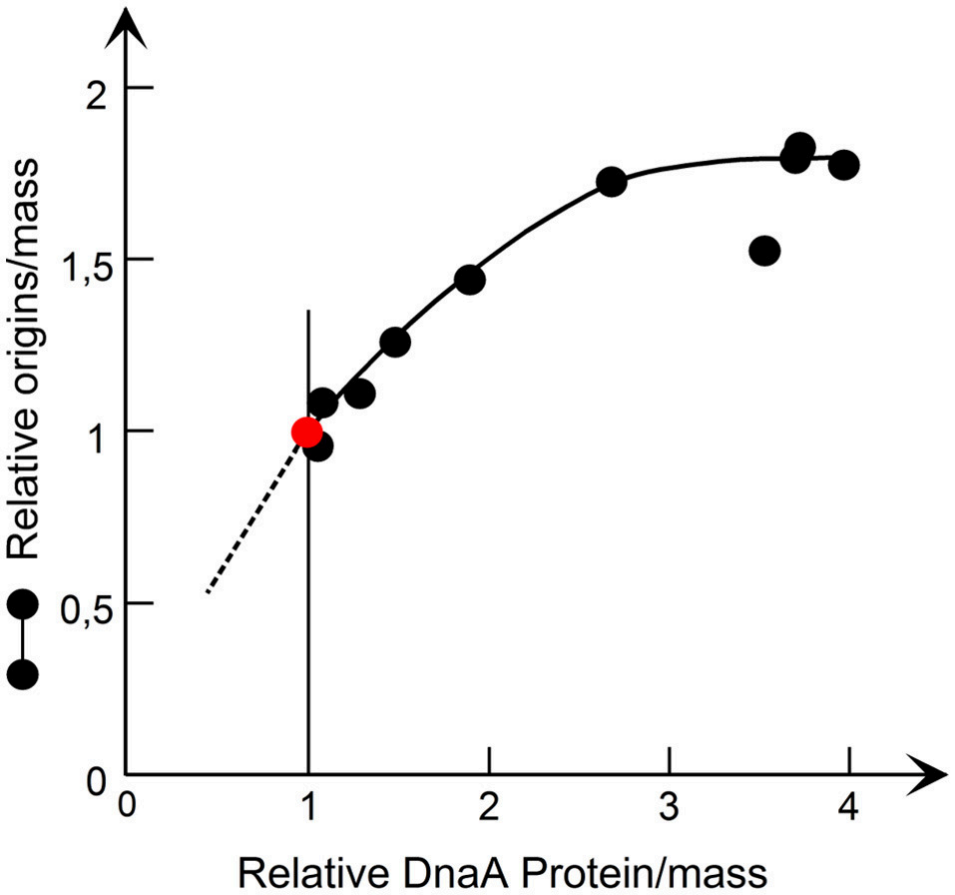
The origin/mass responds to an increase in DnaA protein. The DnaA protein was synthesized from a *lacP* controlled *dnaA* gene and origins/mass was determined by flow cytometry. The data are redrawn from Figure 4 in Atlung and Hansen (1993, Copyright, American Society for Microbiology).

The first evidence that DnaA was not just essential for the initiation process but could be the initiator regulating initiation came from the studies of *dnaA*(Ts) mutants at permissive temperature which showed that initiation mass increased with increasing growth temperature (Hansen and Rasmussen, 1977; Tippe-Schindler et al., 1979). Further support for DnaA as the initiator was provided by the *dnaA*cos mutant (a pseudorevertant of *dnaA46*) which has a decreased initiation mass at low temperature (Kellenberger-Gujer et al., 1978). The hypothesis that DnaA was the initiator was disputed after the first studies of strains carrying DnaA overproducing plasmids since they did not show an increase in DNA per mass as expected from a decreased initiation mass (Churchward et al., 1983). An analysis measuring also the actual concentration of *oriC* DNA per mass (Atlung et al., 1987) revealed that initiation was stimulated in cells overproducing DnaA protein, but the replication speed was decreased so much that there was virtually no increase in DNA per mass. A later analysis where we used p*lac* controlled expression to increase the DnaA protein concentration from 1.2- to 4-fold the normal (Atlung and Hansen, 1993) showed that within a limited range the origins per mass responded to the DnaA protein concentration (see Figure 4) showing that DnaA protein is indeed the limiting factor for initiation. Also in these experiments replication speed is decreased inversely with the higher DnaA protein concentration. Supporting this finding are the following experiments: (1) where wild type DnaA protein was induced to different subnormal levels in a *dnaA46* mutant at 42°C which showed low DNA per mass (Løbner-Olesen et al., 1989); (2) lowering the expression in a *dnaA*^+^ strain by interfering with the expression of the messenger of the chromosomal *dnaA* gene using the tCRISPRi system (Si et al., 2017); and 3. using a slowly growing *dnaA204* mutant where the DnaA protein concentration was significantly reduced, due to instability of the protein, causing an increase in the initiation mass and thus a decrease in origin to mass ratio (Torheim et al., 2000).

## The DnaA protein structure

The DnaA protein has four domains (Messer et al., 1999). The domain structure was first suggested by the comparison of the *E. coli* and *B. subtilis* proteins (Ogasawara et al., 1985) which showed that there is a moderately conserved N-terminal region and a more highly conserved large C-terminal region separated by a region of highly variable sequence and length. More closely related proteins show a high conservation of the N- and C-terminal domains—e.g., the Enterobacterial proteins have virtually identical sequences in these domains—but many amino acid substitutions, deletions and insertions in the variable region (Skovgaard and Hansen, 1987; Skovgaard, 1990). The C-terminal region contains domains III and IV, with an AAA+ family ATPase motif and the DNA binding domain respectively (Messer et al., 1999). It took a long time after the initial purification of the DnaA protein (Chakraborty et al., 1982; Fuller and Kornberg, 1983) before the first physical structures of DnaA were obtained and as yet no structure of the full length protein has been reported. The structure of domain IV from *E. coli* was determined by NMR and (Obita et al., 2002) and a year later the crystal structure of domain IV in complex with a DnaA-box was obtained (Fujikawa et al., 2003). This confirmed that the DNA binding is mediated by a combination of a basic loop and a helix-turn-helix motif. The structure of domain III has only been obtained from *Aquifex aeolicus* (Erzberger et al., 2002). When bound to ATP, but not to ADP, DnaA forms a super-helical structure with four monomers per turn (Erzberger et al., 2006; see Mott and Berger, 2007; for review and for the best figure). The structure of domain I has been determined for *E. coli* by NMR (Abe et al., 2007) and from three other bacterial species (see Zawilak-Pawlik et al., 2017 for review).

## Function of the different domains

**Figure 5:**
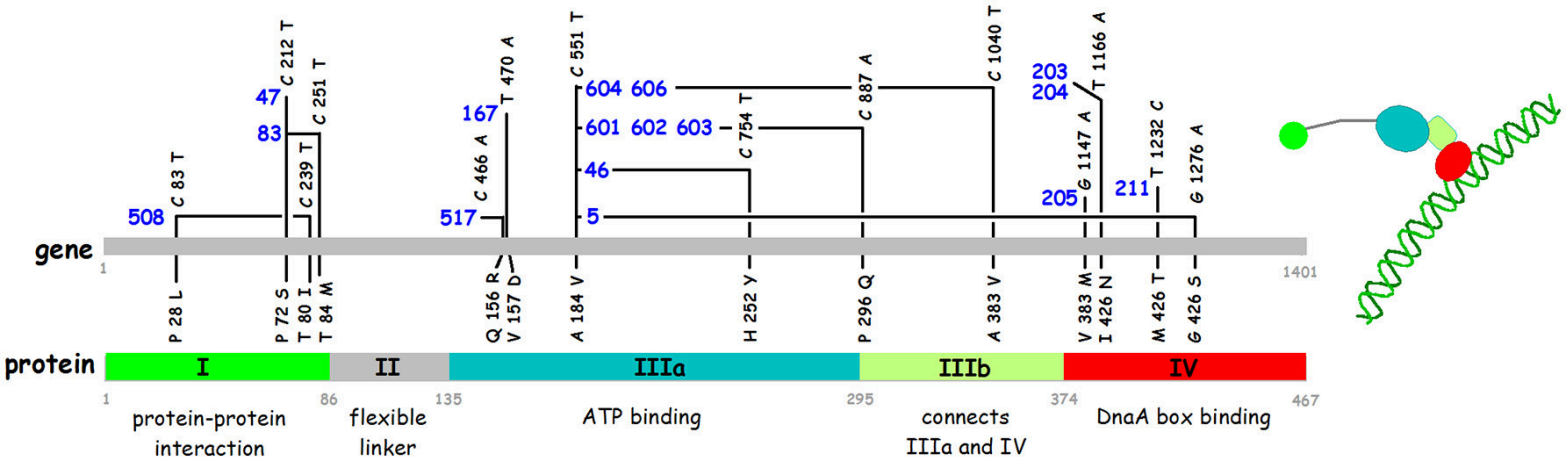
Nucleotide and amino acid alterations in the *dnaA* gene of sixteen temperature sensitive mutants. The mutant numbers are in blue. References for the isolation of the different mutants are: *dnaA508, dnaA517* (Wechsler and Gross, 1971); *dnaA47* (Kuempel, 1969); *dnaA46, dnaA83* (Kohiyama et al., 1966); *dnaA167* (Abe and Tomizawa, 1971); *dnaA601, dnaA602, dnaA603, dnaA604, dnaA606* (Sevastopoulos et al., 1977); *dnaA203, dnaA204, dnaA205, dnaA211* (Beyersmann et al., 1974). The sequences of 12 of these mutants are described by Hansen et al. (1992). The four mutations *dnaA47, dnaA83, dnaA517*, and *dnaA603* were sequenced using PCR fragments obtained from the original mutant strains (this work). The domains in the DnaA protein and the borders between them are shown in the lower part of the figure.

The first indication that each of the domains is essential for the activity of the DnaA protein came from the mapping and sequencing of the classical *dnaA*Ts mutants (Hansen et al., 1984, 1992), see Figure 5 for position of the mutations and their amino acid changes. Since then many other *dnaA* mutants have been isolated both by different selections and by screening of *in vitro* generated mutants (e.g., reviewed in Erzberger et al., 2002; and additional mutants described in the following citations: Felczak and Kaguni, 2004; Asklund and Atlung, 2005; Felczak et al., 2005; Chodavarapu et al., 2013).

The C-terminal domain IV is alone responsible for sequence specific binding to double stranded DNA and the classical *dnaA*Ts mutations mapping here reduce the binding (Roth and Messer, 1995).

Domain III contains the AAA+ ATPase motif and is responsible for nucleotide binding (Sekimizu et al., 1987). DnaA binds both ATP and ADP with similar affinities, K_D_ 10–30 nM and 60–100 nM respectively (Sekimizu et al., 1987; Kawakami et al., 2006; Keyamura and Katayama, 2011), and the ATP form is required for the normal control of initiation of replication (Sekimizu et al., 1987). The ATP form facilitates cooperative binding to weak DnaA boxes (Margulies and Kaguni, 1996; Speck et al., 1999; McGarry et al., 2004), is necessary for unwinding of the DUE (Duplex Unwinding Element) in *oriC* (Sekimizu et al., 1987), and for binding to single stranded (ss) DNA in the DUE (Speck and Messer, 2001; Ozaki and Katayama, 2012). Domain III binds the ss DNA directly through specific amino acids, e.g., V211 and R245 (Ozaki et al., 2008; Duderstadt et al., 2011). The terminal part of Domain III also contains a sequence patch for interaction with the membrane (Garner and Crooke, 1996) which stimulates release of the nucleotide from DnaA (Sekimizu and Kornberg, 1988). Except for *dnaA167*, and possibly also *dnaA517*, all the other classical Ts *dnaA* mutants that map in domain III carry the same mutation, i.e., the A184V change close to the P-loop of the Walker A ATP binding motif and four different secondary mutations (Figure 3), and the DnaA46 protein has been shown to be defective in nucleotide binding (Hwang and Kaguni, 1988; Carr and Kaguni, 1996).

Domain II is thought to be a flexible linker connecting domains I and III. All parts of domain II (between residues 78 and 136) can be deleted without major effects on function as long as the linker is 21–27 amino acids long (Nozaki and Ogawa, 2008) and the entire YFP sequence can be inserted without any effect on function (Nozaki et al., 2009a).

Domain I has several functions in relation to protein-protein interactions (see Zawilak-Pawlik et al., 2017 for review). Domain I is responsible for dimerization of DnaA protein (Weigel et al., 1999). The dimerization is essential for the function of the protein in initiation of replication as deletion of the first 24 amino acids renders the protein inactive in complementation of the *dnaA46* mutant (Weigel et al., 1999). Residues 24–86 are essential for interaction with DnaB, but recruitment of DnaB at *oriC* also requires the first 23 amino acids (Seitz et al., 2000). The DiaA (*D*naA *i*nitiator *a*ssociating protein) also interacts with domain I. The *diaA* gene was identified in a selection for suppressors of the *dnaAcos* mutant that over initiates (Ishida et al., 2004). The DiaA protein is not essential, but stimulates initiation of replication *in vitro*, and a *diaA* null mutant is slightly impaired in initiation and shows moderate asynchrony (Ishida et al., 2004). The DiaA protein is a tetramer (Keyamura et al., 2007, 2009). The amino acids in DnaA involved in dimerization and in DiaA/DnaB interaction are located on opposite faces of the 3 D structure of domain I, therefore the DiaA tetramer protein can act as a connector for more distantly located DnaA dimers and monomers.

## Autoregulation of DnaA protein synthesis

The first indication that the *dnaA* gene is autoregulated came from the studies of *dnaA*(Ts) mutants. DnaA protein continues to be synthesized at non-permissive temperatures where it is inactive, but in some mutants it can be reactivated when brought back to a permissive growth temperature (Hanna and Carl, 1975; Messer et al., 1975). This reversible initiation capacity, which could be observed in the absence of protein synthesis at the permissive temperature, was higher than expected from the mass increase of the culture and suggested that the DnaA protein synthesis was derepressed at the high growth temperature. These findings led to a detailed study of the *dnaA46* mutant at permissive, intermediate, and non-permissive growth temperatures (Hansen and Rasmussen, 1977). The results obtained in this study suggested that the DnaA46 protein became less and less active—the higher the growth temperature—resulting in gradually lower DNA and origin concentrations at the intermediate temperatures. In parallel with the decrease in DnaA protein activity an increase in initiation capacity was observed. Based on these findings Hansen and Rasmussen (1977) proposed that the DnaA protein had a positive role in initiation of replication and a negative role in its own synthesis.

**Figure 6:**
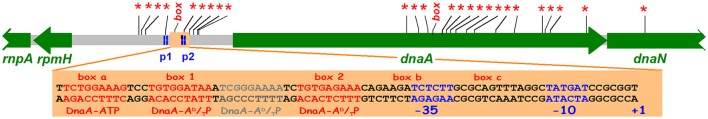
The *dnaA* region with GATC sites (^*^) and consensus DnaA boxes (box). The position of *dnaA* promoters p1 and p2 are indicated (Hansen F. G. et al., 1982). The light orange rectangle shows the p2 promoter and potential DnaA boxes upstream of and overlapping the promoter. box1, box2 and the region in between are protected both by DnaA-ADP and DnaA-ATP, the boxes a, b, and c require DnaA-ATP (Speck et al., 1999). The CCGCGG sequence upstream of the +1 position is a Travers box (Travers, 1980).

The *dnaA* promoter region and the *dnaA* gene was sequenced (Hansen E. B. et al., 1982; Hansen F. G. et al., 1982) and two promoters giving transcripts entering the *dnaA* gene were identified. The DnaA promoter region carries 9 GATC sites within 225 bp and between the two promoters a sequence homologous to repeats (DnaA-boxes) in the *oriC* region was identified (box 1 in Figure 6). Both promoters are negatively regulated by DnaA protein (Atlung et al., 1984, 1985; Braun et al., 1985; Kücherer et al., 1986). In these studies the *dnaA* transcription was found to be increased up to 5-fold in *dnaA*Ts mutants at non-permissive temperature and to be repressed 4- to 5-fold by overproduction of DnaA protein. The DnaA-box is essential for the autoregulation of the *dnaA* gene (Atlung et al., 1984, 1985; Braun et al., 1985). A closer look at the sequence of the *dnaA2p* promoter region reveals interesting features. Two GATC sites are present in this promoter, one in the−10 sequence and the other in the −35 sequence, and methylation enhances transcription 2-fold from this promoter both *in vivo* and *in vitro* (Braun and Wright, 1986). Furthermore, DnaA protein binds with high affinity to sequences upstream of the *dnaA2p* promoter (Speck et al., 1999). This *in vitro* study showed that DnaA-ATP as well as DnaA-ADP binds to what they call DnaA box 1 and DnaA box 2, whereas a box they call “DnaA box a” that has two misfits to the consensus sequence (see below) only binds DnaA-ATP. From a comparison of the *dnaA2p* promoter region with the *mioC* promoter region (see below) as well as from a closer look at the *in vitro* binding data (Speck et al., 1999) we suggest that the region act as having four closely spaced DnaA boxes upstream of the *dnaA2p* promoter region one of which requires DnaA-ATP for binding (Figure 6) and two sites, box b and box c with no homology to DnaA boxes, which also requires DnaA-ATP. The DnaA-ATP form is required for repression of both promoters *in vitro* (Speck et al., 1999) in accordance with the location of the different kinds of DnaA-boxes.

## Other features of *DnaA* gene expression

### Transcription

The *dnaA* promoter region is fairly strong, giving transcription corresponding to 50% of the *tet* promoter in pBR322 (Atlung et al., 1984). The *dnaA* gene proximal promoter p2 is approximately 3-fold stronger than the p1 promoter (Atlung et al., 1985; Kücherer et al., 1986; Chiaramello and Zyskind, 1990). *dnaA* transcription has been reported to be growth rate regulated decreasing with decreasing growth rate (Chiaramello and Zyskind, 1990; Polaczek and Wright, 1990). The p2 promoter carries a discriminator “Travers box” (Travers, 1980) downstream of the −10 sequence and transcription is, like that of the growth rate regulated *rrnP1* promoter, almost completely inhibited by high ppGpp levels.

### Translation and mRNA stability

The coding sequence of *dnaA* has a GUG start codon and a rather poor ribosome binding site (GGAG). The efficiency of translation initiation in mRNA with a SD sequence is 2- to 3-fold lower with a GUG start codon relative to an AUG start codon (Reddy et al., 1985; Donnell and Janssen, 2001). That this is similar for the *dnaA* mRNA is supported by the finding that the *dnaA508cos* mutant carrying a GUG to AUG mutation has an increased DnaA protein level sufficient to give auto-suppression of the temperature sensitivity (Eberle et al., 1989). This is similar to suppression of the temperature sensitivity obtained by having a *dnaA*(Ts) gene on pBR322 derived plasmids and on the chromosome (Hansen et al., 1992; Nyborg et al., 2000).

In addition to the low translation frequency the production of DnaA protein from the mRNA is limited by a short mRNA half-life. The chemical half-life is in the low range, in minimal medium it was amongst the shortest 1% of all mRNAs (1/3 of the mean half-life, Bernstein et al., 2002).

The wild type DnaA protein is stable (Atlung and Hansen, 1999) and is present in K-12 at ~200 monomeric molecules per *oriC* (Hansen et al., 1991b). Therefore, the cell has to produce 100 new molecules per *dnaA* gene per generation. The above characteristics of DnaA expression, moderately high transcription rate, low translation initiation frequency and highly unstable mRNA we estimate to give on the average one protein molecule per transcript. These are the features needed to ensure a well-regulated production of protein over the cell cycle that responds smoothly to regulatory changes in transcription initiation (see **Figure 8**).

## DnaA protein at different growth rates

DnaA protein concentration in several different *E. coli* K-12 strains and in S*almonella typhimurium* was determined by immunoblot analysis and found to be very similar in all these strains (Hansen et al., 1991b). This study furthermore showed that the concentration of DnaA protein in all the strains was the same at all growth rates (doubling times between 150 and 22 min). Similar studies using four different primary DnaA antibodies gave the same results (Herrick et al., 1996). The constant DnaA protein concentration might intuitively be in conflict with increasing levels of mRNA with increasing growth rate. But you have to consider two factors: (1) the time available for protein synthesis, i.e., the generation time, (2) the translation efficiency, i.e., the number of proteins produced per mRNA at the different growth rates (see Table 1). If the translation efficiency is the same at 30 and 60 min doubling times, you will need twice as many mRNAs/mass to produce the same number of protein molecules/mass in the faster growing cells. It has been calculated that the average mRNAs are translated more efficiently in faster growing cultures, close to proportionality between number of translations and growth rate (Bremer and Dennis, 1996). The *dnaA* mRNA is, however, very different from the average mRNA in translation efficiency, we estimated it to be one translation per mRNA (see above) compared to the 30–90 translations for the average mRNA. It is therefore likely that *dnaA* mRNA efficiency might be more similar at different growth rates.

**Table 1:** DnaA protein production at different growth rates.

		**Generation time (min)**			
**Row**	**Entity**	**100**	**60**	**30**	**Reference/derivation**
1	Relative total RNA/mass	0,6	0,74	1	Bremer and Dennis, 1996
2	% mRNA of total RNA	2	2	2	Bremer and Dennis, 1996
3	Relative total mRNA/mass	0,6	0,75	1	From rows 1 and 2
4	Average no of translations per mRNA	27	33	71	Bremer and Dennis, 1996
5	Relative *dnaA* mRNA/total RNA	0,27	0,37	1	Chiaramello and Zyskind, 1990
6	Relative *dnaA* mRNA/mass	0,45	0,5	1	from rows 1 and 5
7	Relative DnaA per mass	1	1	1	Hansen et al., 1991b

## Initiator titration

The first studies on titration of DnaA protein by DnaA boxes *in vivo* were conducted looking at derepression of the *dnaA* gene by DnaA boxes from *oriC*—*mioC* and the *dnaA* promoter region using a single copy *dnaA-lacZ* fusion (Hansen et al., 1987) and lead to the formulation of the initiator-titration model for control of initiation of replication (see below, Hansen et al., 1991a).

This was followed by two studies aimed at identifying other high affinity DnaA binding sites on the chromosome. In the first only one fragment was obtained and analyzed (Kitagawa et al., 1996). It carried what was later named the *datA* (DnaA titration) locus. The *datA* locus contains two consensus DnaA boxes and four 1-misfit boxes (see **Figure 14**) but was found to titrate much more DnaA protein than the *oriC mioC* region (Kitagawa et al., 1996). The other study, using a fishing method to isolate high affinity DNA fragments, identified five loci (in addition to *oriC*) including *datA* and the *mutH* promoter (Roth and Messer, 1998) that was later found in the search for DARSs (DnaA Reactivating Sequences, see below; Fujimitsu and Katayama, 2004). The *in vivo* titration ability—effect on *dnaA* transcription—of these other four loci has not been studied.

The consensus DnaA box TTWTNCACA was suggested by Schaper and Messer (1995) based on binding of purified DnaA protein to 21 bp oligonucleotides. There are 307 of these consensus DnaA boxes on the *E. coli* chromosome. The DnaA box quality depends on the base in the fifth position and on the neighboring base sequence. Oligonucleotides which carried a DnaA box of the R1-R4 type (TTATCCACA) and had the neighboring bases as present in *oriC* were very efficient in binding the protein (K_D_ 10^−9^ M). Similar experiments were carried out using 17 bp double stranded oligonucleotides with 6 bp upstream and 2 bp downstream of the four consensus boxes starting with TTAT and the *mioC* (R5) box TTTTCCACA as well as with 104 bp PCR fragments with identical sequences except for the 8 different consensus boxes in the middle of the PCR fragment (Hansen et al., 2006). In the boxes starting with TTAT a C in the fifth position (R1-R4 type) is better than A (R2) or G which is better than T. In the boxes of the *mioC* type starting with TTTT, C in the fifth position is much better than the other possibilities, which show very weak or unspecific binding. Plasmids carrying the *mioC* promoter with the 8 different consensus boxes gave varying degrees of titration of DnaA protein that was in good accordance with the *in vitro* determined relative binding affinities, and the degree of repression of transcription from the *mioC* promoter also varied as expected from the binding affinities.

**Figure 7:**
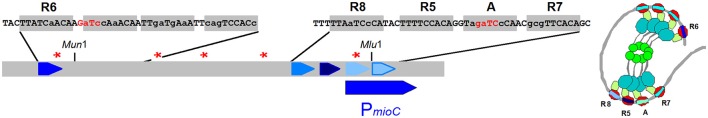
The five experimentally verified DnaA boxes in the *mioC* region are named above the sequence boxes (Hansen et al., 2007) but also potential box sequences to the right of the R6 box are indicated. GATC sites are indicated with red stars (or red letters). To the right is a speculative view of DnaA protein bound to the DnaA-boxes in the *mioC* region (see Figure 5 for domain colors).

As noted above the *mioC* promoter region carries one consensus DnaA box and the *oriC* region carries three boxes, which in the gel shift assays were found to be very efficient binders. Nevertheless, the plasmid carrying the *mioC* promoter region is as efficient in titrating DnaA protein as a plasmid carrying an inactivated *oriC* region (Hansen et al., 2006). This study also demonstrated that sequences ~100 bp upstream of the promoter contributed to efficient titration and thus derepression of the *dnaA-lacZ* promoter. An even more detailed study (Hansen et al., 2007) led to definition of DnaA boxes in the *mioC* region with several misfits (Figure 7). The number of DnaA proteins per origin has been determined to be ~200 monomers in several different *E. coli* K-12 derivatives growing at different growth rates (Hansen et al., 1991b). The plasmid with the *mioC* promoter region increases *dnaA* gene expression 1.4-fold corresponding to 80 DnaA monomers and as the pBR322 derived plasmid carrying the *mioC* promoter region is present in ~10 copies/*oriC* (Atlung et al., 1999) it follows that each *mioC* promoter region titrates approximately eight DnaA monomers. Similar calculations for the titration caused by the *dnaA* promoter region (Hansen et al., 1987) suggest that the *dnaA* promoter region titrates 3–4 DnaA proteins.

The presence of plasmids carrying DnaA boxes affects initiation of replication leading to increased initiation mass in parallel with the increased *dnaA* gene expression (Christensen et al., 1999; Morigen et al., 2003).

As mentioned above the *datA* region that carries two consensus DnaA boxes surrounded by closely spaced boxes with up to several misfits (see **Figure 14**) titrates large amounts of DnaA protein and plasmids carrying these boxes are difficult to handle in wild type cells.

## GATC sites, SeqA protein, and the eclipse period

The average density of the GATC sequence in a DNA sequence containing equal amounts of the four bases is one in 256 bp. The density of GATC sites in the *oriC* sequence and the *dnaA* promoter sequence is approx. ten times higher (Figures 3, 6). Unmethylated plasmids carrying the *oriC* or the pBR322 origin cannot transform Dam^+^ strains, and fully methylated plasmids cannot transform a Dam^−^ strain. In both of these cases the plasmids will become hemimethylated upon replication in the recipient cell, therefore it was concluded that full methylation was essential for the establishment of the plasmids (Russell and Zinder, 1987). The *oriC* and the *dnaA* regions become hemimethylated after initiation of replication and stays hemimethylated (are sequestered) for a considerable time period (1/3 and 1/5 of a doubling time, respectively) while regions with few GATC-sites become methylated within 1–2 min (Lark, 1968; Campbell and Kleckner, 1990).

The SeqA protein binds to closely spaced hemi-methylated GATC sites (Brendler et al., 2000) and the *oriC* and *dnaA* promoter regions contains several closely spaced GATC sites (see Figures 3, 6). These regions were “sequestered” in much shorter periods when the *seqA* gene was mutated or absent (Lu et al., 1994; von Freiesleben et al., 1994). A flow cytometric study of the re-initiation kinetics in several *dnaA*(Ts) mutants which had been initiation aligned at high temperatures showed that after the immediate initiation of one round of replication at the permissive temperature there was a constant number of origins until a second initiation took place (Hansen, 1995). This period, the eclipse, was studied in more detail in strains producing normal, less than normal, and higher than normal Dam-methylase (von Freiesleben et al., 2000a). These experiments showed clearly that the length of the eclipse (sequestration) period was inversely related to the cellular level of Dam-methylase.

## The initiation cascade

Flow cytometry studies of wild type and *dam* mutant cells using synchronous cultures obtained by the baby cell machine (Løbner-Olesen et al., 1994) gave the experimental evidence for the initiation cascade as a model for how *E. coli* containing multiple origins initiates all of these once and only once per cell cycle (Hansen et al., 1991a). The cascade states that in wild type cells initiation of one origin will lead to release of DnaA protein bound to that origin, these DnaA molecules cannot bind to the two new *oriC* sequences due to the sequestration of the hemimethylated DNA by the SeqA protein. Therefore, the level of unbound DnaA protein is raised and will promote initiation at another “old” fully methylated origin. Thus, initiation will continue until all origins have initiated. To prevent reinitiation in the same cell cycle the availability of unbound initiation competent DnaA protein must be lowered before the end of the eclipse period.

The initiation cascade also explains the lack of incompatibility between minichromosomes and the chromosome despite the fairly high copy number of the minichromosomes (average 8–10 copies per chromosomal *oriC*; Løbner-Olesen et al., 1987). Before the isolation and characterization of the first minichromosomes it was expected, based on the observations with plasmids, that there would be incompatibility between the chromosomal and minichromosomal *oriC*s, i.e., that the presence of minichromosomes would inhibit initiation of chromosomal replication. Baby cell experiments showed that the minichromosomes initiate at the same time in the cell cycle as the chromosome (Leonard and Helmstetter, 1986), and flow cytometric analysis showed that the presence of a minichromosome only has a very small effect on the synchronous initiation of 4 or 8 chromosomal origins in fast growing cells (Løbner-Olesen and von Freiesleben, 1996; Skarstad and Løbner-Olesen, 2003). In contrast DnaA boxes carried on plasmids like pBR322 that replicate randomly during the cell cycle disturbs the initiation cascade leading to asynchrony (Christensen et al., 1999; Morigen et al., 2003).

## Synthesis of DnaA protein during the cell cycle

Transcript analysis using an initiation aligned *dnaC2* mutant showed that after 30 min at 40°C transcription of the *mioC* gene was completely shut down, *dnaA* transcription was halved and *gidA* transcription was virtually unaffected (Theisen et al., 1993). This suggests that DnaA protein synthesized during the incubation of the culture at 40°C will be filling the DnaA boxes in the *mioC* promoter region effectively repressing all transcription, while the *dnaA* promoter will be less affected since it can only be partially repressed. The *gidA* promoter, that has no DnaA boxes, is not affected at the high temperature. Soon after initiation at the permissive temperature, there was virtually no transcription from the *dnaA* promoter and only a little from the *gidA* promoter whereas *mioC* transcription was restored after a few minutes. One minute after initiation the *dnaA* gene is replicated and the *dnaA* promoter region will become hemimethylated and sequestered by SeqA for a considerable period (~10 min; Campbell and Kleckner, 1990; Lu et al., 1994) inactivating the two promoters. The *gidA* promoter is located close to the sequestered *oriC* and is probably partially co-sequestered with the *oriC* region for some time. The *mioC* promoter is not sequestered and repression will be reduced by decreased DnaA activity due to titration and RIDA as replication proceeds. The findings obtained with the *dnaC* aligned system was confirmed using synchronous cultures obtained with the baby cell machine (Theisen et al., 1993). A considerable number of non-sequestered genes was also analyzed using these two synchronization techniques (Zhou et al., 1997) but no dramatic changes during the cell cycle were observed.

**Figure 8:**
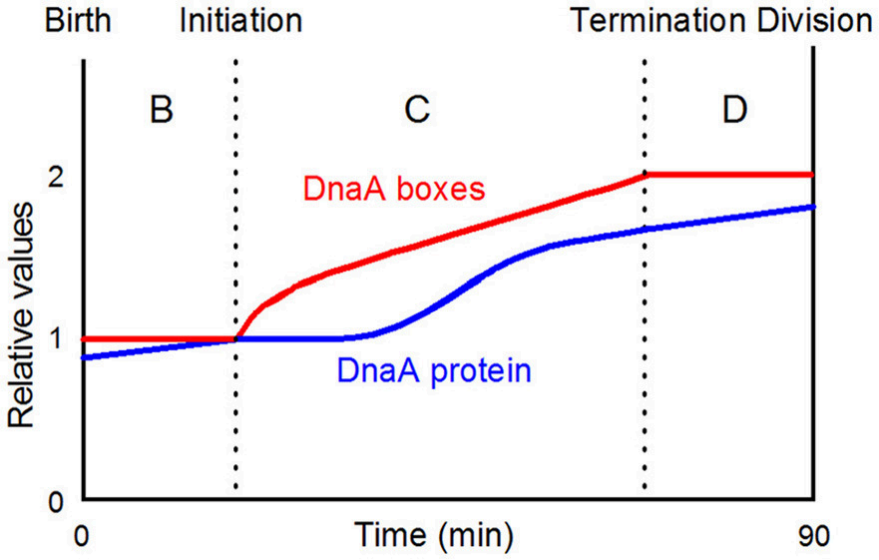
A simplistic view of when initiation takes place in the cell cycle of slowly growing bacteria. The DnaA protein curve is drawn on the basis of the data presented by Theisen et al. (1993). DnaA-boxes with any distribution will be doubled during the C-period; the actual distribution of all consensus DnaA-boxes and selected groups of consensus boxes are presented in Figure 10; the figure is redrawn from Figure 2A in Herrick et al. (1996, Copyright, John Wiley and Sons).

The *dnaA* transcription data presented above (Theisen et al., 1993) allow us to speculate about the relative numbers of DnaA protein and DnaA boxes during the cell cycle in the simple situation of a slowly growing culture. Assuming that DnaA protein is synthesized in proportion to the DnaA messenger we can predict how the number of DnaA proteins increases during a cell cycle (Figure 8). There is a period before initiation where *dnaA* messenger and thus protein is accumulated slowly due to autorepression. Soon after initiation the *dnaA* promoter region is sequestered and both *dnaA* promoters are inactivated. When the *dnaA* promoters are released from sequestration following methylation, the DnaA protein synthesis rate will be high, due to a low concentration of DnaA protein relative to titrating DnaA boxes. The level of DnaA-ATP, that is the active form repressing the *dnaA* promoter, will also be low, due to RIDA and DDAH (see later).

## Models for control of initiation of chromosome replication

The Replicon Model (Jacob et al., 1963) states that an initiator structure has to be build to interact with the origin to initiate replication. At initiation new origins are synthesized and the initiator structure is destroyed or at least prevented from reinitiating at newly synthesized origins. New initiator structures have to be constructed/reformed before initiation can take place in the following cell cycle. Basically such a model is valid but at the time it was presented the molecular basis for the initiator structure could not be addressed.

A model which attracted a lot of attention at these early times was the Inhibitor Dilution Model (Pritchard et al., 1969). It postulates that a fixed amount of messengers coding for inhibitor molecules are synthesized in a burst soon after initiation. These inhibitors would be diluted during growth of the cell and when the concentration reaches a threshold allow initiation to take place followed by the burst of new inhibitors. The molecular mechanism for how to synthesize a “fixed” amount of inhibitor after initiation was not discussed. This model inspired formulation of new models for how initiation in *E. coli* could be controlled. The inhibitor dilution model has later been formally rejected by Margalit and Grover (1987).

**Figure 9:**
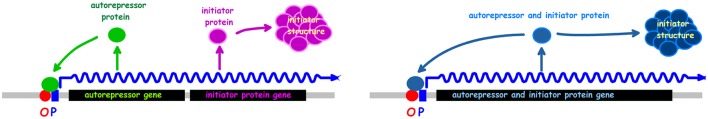
The autorepressor model. The left drawing presents the model in its original formulation (Sompayrac and Maaløe, 1973). The right drawing shows an alternative where the autorepressor and initiator is the same protein.

The autorepressor model (Sompayrac and Maaløe, 1973) hypothesizes the existence of an operon with two genes, one coding for a repressor protein, the other for an initiator protein (Figure 9). The repressor interacts with an operator in the promoter and represses its own synthesis to keep the repressor concentration constant and thus the production of initiator molecules constant. The model proposes that the initiator molecules build an initiator structure which will be ready to initiate when the cell reaches its initiation mass and will be destroyed at initiation. After initiation the cell can start building a new initiator structure which can be used to initiate the following replication cycle. An analysis of the model using a stochastic approach suggested that the synthesis of 250 initiator molecules shows the best fit to the experimental observations in a culture synchronized with the baby cell machine (Clark and Maaløe, 1967). This number is surprisingly close to the number of DnaA proteins per *oriC* found in several *E. coli* strains (Hansen et al., 1991b). The autorepressor model has been tested in a very detailed theoretical study by Margalit et al. (1984) in which it was concluded that the model could be valid provided that the location of the operon is close to *oriC*, that the promoter is moderate to strong, translation efficiency is low, and that the repressor binds with a moderate affinity (K_D_ ~10^−9^ M); the *dnaA* gene and DnaA protein fulfills all of these requirements.

The model published by Mahaffy and Zyskind (1989) was based on data stating that the DnaA protein concentration increases at increasing growth rates (Chiaramello and Zyskind, 1989). This is at variance with the now generally accepted invariance of DnaA protein concentration in K-12. It should be noted that Mahaffy and Zyskind (1989) had to use different nucleotide bound forms of DnaA to get the model to fit with the different DnaA concentrations at different growth rates.

The Initiator Titration Model, which we will cover in some detail, was first presented at the EMBO workshop in Segovia, Spain in 1987 and it did not change before it was published (Hansen et al., 1991a).

The model hypothesizes that:

1. *The initiator—DnaA protein—has a high affinity for specific sites—DnaA boxes on the chromosome*.
2. *The initiator—DnaA protein—has a lower affinity for sites involved in the formation of the initiation complex*.
3. *DnaA protein bound to DnaA boxes is released (pushed off) by replication*.
4. *A newly replicated origin is refractory to initiation for the period it takes to regain a normal topology*.

**Figure 10:**
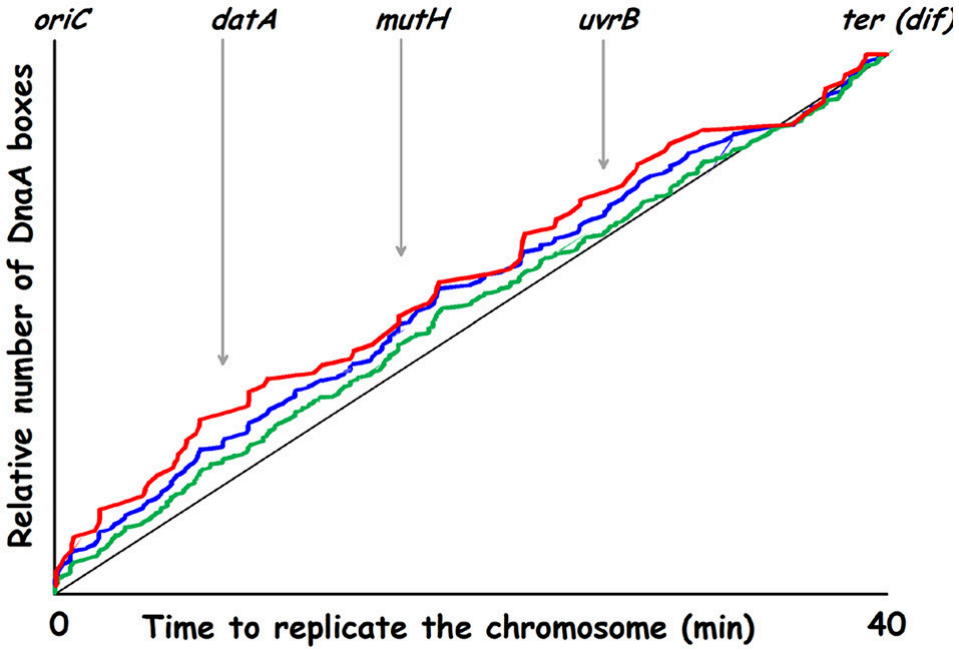
Replication time for DnaA boxes on the K-12 chromosome. Green curve: 307 consensus DnaA boxes (TTWTNCACA); blue curve: 164 DnaA boxes (TTATNCACA + TTTTCCACA); red curve: 78 DnaA boxes (TTATNCACA where N is either C, A, or G). Genes in or lying close to chromosomal regions involved in nucleotide conversions are indicated.

The model was analyzed by computer simulations using a stochastic approach. A main goal was to find ONE set of parameters to put into *in silico* experiments which could be compared to different *in vivo* experimental data. To this end the comprehensive theoretical study which concluded that the autorepressor model might be a possible model (Margalit et al., 1984) was very helpful. Some of the parameters we have used are presented in Table 2. When we designed parameters for the model we hypothesized that the active form of DnaA protein was a tetramer and most often we used 75 DnaA-boxes distributed on the chromosome, usually with a higher occurrence of DnaA-boxes close to the origin. The actual consensus DnaA box distribution on the chromosome is presented in Figure 10.

**Table 2:** Selected parameters used in the Initiator Titration Model.

**Input variables**		**Calculated numbers**	
τ	Doubling time	V	Cell volume
C	Replication time	T	Age of cells
D	Time from termination to cell division	T_n_'s	Replication fork positions
A_i_	Number of DnaA molecules required for initiation	n	DnaA boxes per growing cell
K_D_	Dissociation constant for DnaA-DnaA box interaction	G	Genome equivalents per cell
k*_*dnaA*1*p*_*	Promoter constant *dnaA1p* (3)^a^	g	*dnaA* genes per cell
k*_*dnaA*2*p*_*	Promoter constant *dnaA2p* (15)^a^	A	DnaA proteins per cell
k*_*dnaAcp*_*	Promoter constant *dnaA* constitutive component (3)^a^	m	*dam* genes per cell
k*_*dam*_*	Promoter constant *damP* (0.5)^a^	δ	Fraction of time the *dnaA* promoters are open (calculated using K_D_, A, n, V)

a *The numbers in parentheses are relative promoter activities*.

In the computer experiments we started with one predefined unit baby cell that was allowed to run through 50 generations where one randomly chosen daughter was discarded. After 50 generations randomly selected daughters (baby cells) were saved in an array until arriving at a chosen cell number (e.g., 1,000 cells). These computer cells can be used directly as a perfectly synchronized culture, or they can be randomized to obtain an exponential culture by starting the baby cells one by one at different times. The computer culture grows and accumulates cells, until the cell number has doubled, then the computer culture is diluted 2-fold.

**Figure 11:**
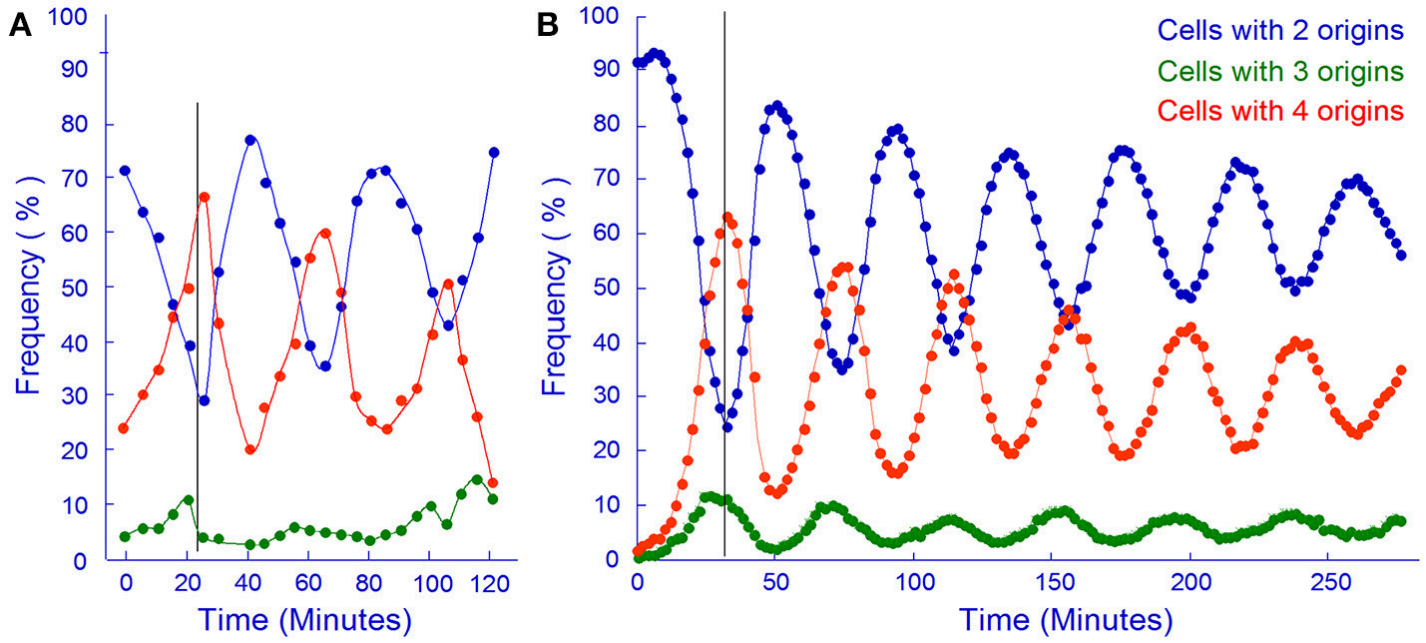
*In vivo* and *in silico* synchronized cultures. **(A)** Samples from a synchronous culture obtained using the baby cell machine were incubated with rifampicin and the frequency of cells containing different numbers of origins were determined by flow cytometry (redrawn from Figure 4A in Løbner-Olesen et al., 1994, Copyright, John Wiley and Sons). **(B)** Same as A but using computer simulation of the Initiator Titration Model to obtain baby cells. The cell size variation in this simulation had a CV of 6.3.

**Figure 12:**
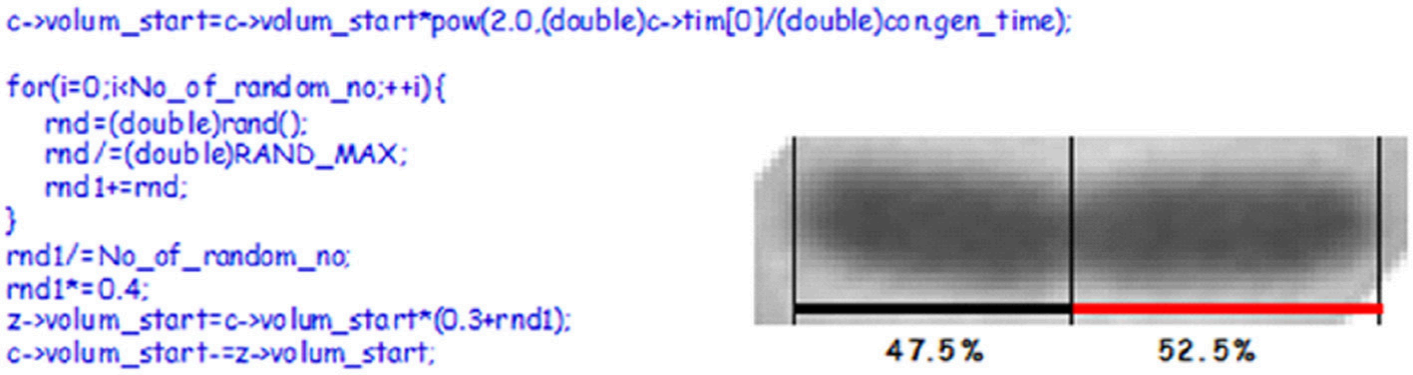
The picture shows a constricted cell of *E. coli* which will divide asymmetrically. The cell length and the position of the constriction was found using a macro written for the Image-Pro Plus software (Nielsen and Hansen, 2010). To the left is C-code showing how we handle cell division in our simulation. c is the starting cell that increases exponentially in size with a constant generation time. At division a sum of random numbers are generated and divided with the number of random numbers. For this calculation we assume that the division take place in the central 40% of the cell (~the multiplication by 0.4), thus one of new cells will inherit 30% of the size of the old cell plus what came out of the random number considerations. If the No_of_random_no is 8 or 13 it will give CVs of 8.3 or 6.3, respectively.

The *in silico* experiment shown in Figure 11B was first carried out with the assumption that the mother cell divided into two equally sized baby cells. To our surprise these simulations resulted in a computer culture behaving incredibly synchronous and that kept the synchrony for a much longer time than should be expected (data not shown). This *in silico* result was the reason why we changed the cell division mode to have a stochastic positioning of the division septum. To illustrate our stochastic approach we present an example where we use experimental data from the literature to address cell size variation after cell division. The cell length of mothers and daughters of slowly growing *E. coli* B/r cells were analyzed (Schaechter et al., 1962; Koppes et al., 1978) and found to exhibit some variation (coefficient of variation, CV, of 8–10%) in the positioning of the septum. Figure 12 demonstrates how we handle every single cell at division.

### Consequences of asymmetric divisions

The synthesis of DnaA protein can be described by the formulas:

(1) $$\begin{array}{r} \frac{dDnaA}{dt}=C_{cell}*C_{promoter} \end{array}$$

*C*_*cell*_ is a component describing the general status of the cell at a given time point:

(2) $$\begin{array}{r} C_{cell}=\;\frac{PSS*g}{G} \end{array}$$

where PSS is the amount of ribosomes, RNA polymerase, and other proteins, as well as nucleotides, amino acids and other small molecules needed to synthesize proteins, RNA, and DNA. The PSS will be proportional to the cell size. The g is the actual number of *dnaA* genes in the cell at any given time in the cell cycle, and G is the actual number of genome equivalents in the cell which will be proportional to all the other genes. And the promoter component:

(3) $$\begin{array}{r} C_{promoter}=k_{\mu }\times \alpha \left(\delta \left(k_{dnaA1p}+k_{dnaA2p}\right)+k_{dnaAc}\right) \end{array}$$

that is growth rate dependent (*k*_μ_), α is 0 if the promoter region is sequestered or 1 if the region is accessible, the *k*_*dnaA*1_ and *k*_*dnaA*2_ are promoter constants, *k*_*dnaAc*_ describes the DnaA protein synthesis that cannot be repressed. For each cell in the computer at each time step as the cell grows the degree of promoter openness (δ) will be calculated from K_D_, the number of DnaA proteins, and the number of DnaA boxes.

Thus if a cell divides asymmetrically one of the babies will become bigger and will therefore contain more PSS, but each baby will be born with the same number of number of *dnaA* genes (g) and the same amount of genome equivalents (G). The net result of the asymmetric division will be that the big daughter synthesizes DnaA protein a little faster than the smaller and therefore will be ready for the next initiation a little faster than the other. The overall result of this is that the two cells have about the same size (but not the same age) at the next initiation.

Today we know much more than we did in 1987, and we have to include the different nucleotide bound forms of DnaA protein (see later). However, the model with its original parameter setting can simulate several experiments as published earlier (Hansen et al., 1991a) and the one shown in Figure 11.

## Recent additional features of regulation of initiation by DnaA

**Figure 13:**
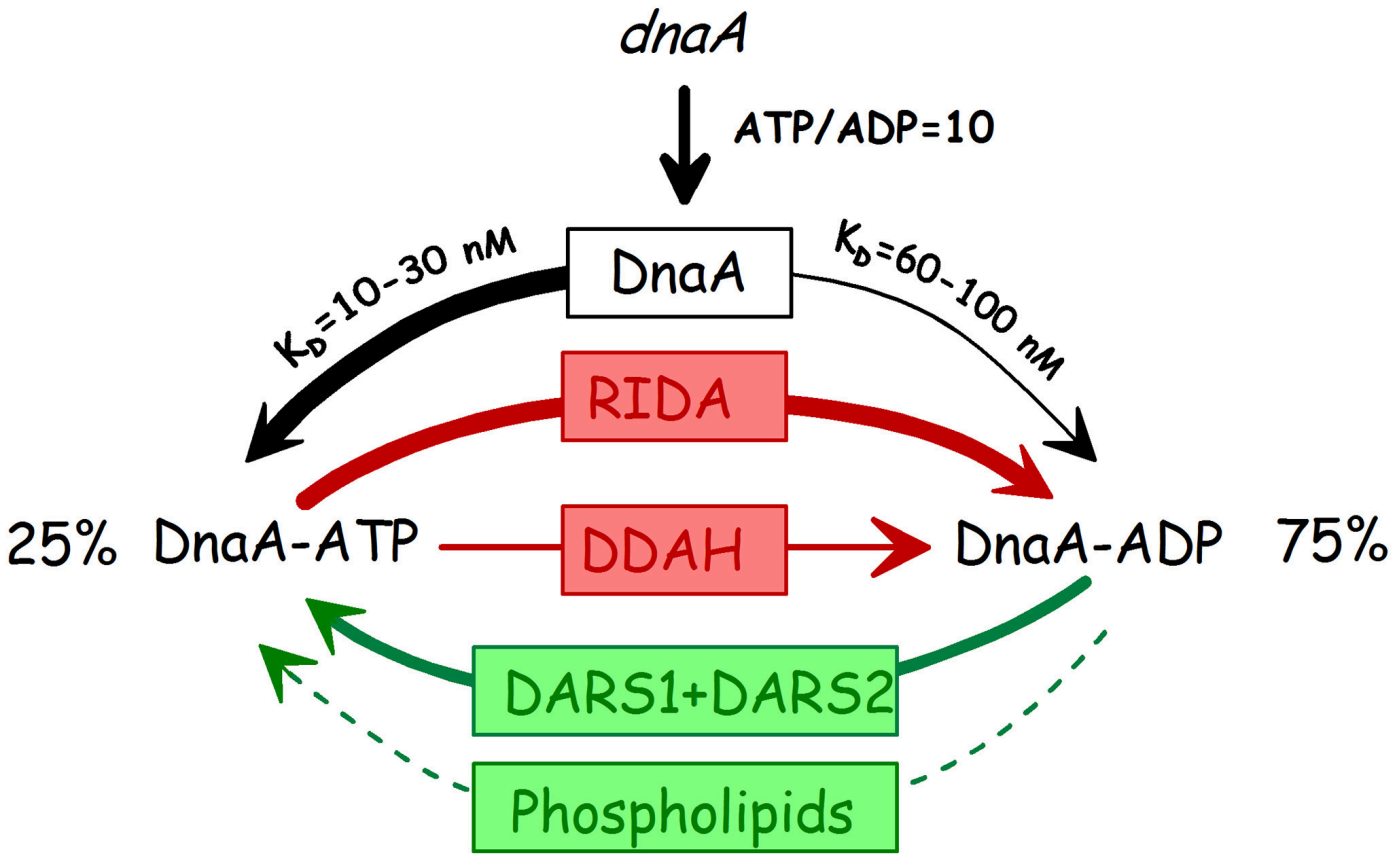
The three states of the DnaA protein. The thickness of the arrows indicate the approximate relative contributions of the different elements to the interconversion of the different forms. Stipled arrow: the relative contribution of phospholipid mediated nucleotide exchange (Sekimizu and Kornberg, 1988; Aranovich et al., 2006, 2015) is unknown. RIDA: Replicative inactivation of DnaA (Katayama et al., 1998; Kato and Katayama, 2001); DDAH: *datA* dependent ATP hydrolysis (Kasho and Katayama, 2013); DARS: DnaA Reactivating sequence (Fujimitsu et al., 2009).

The initiator titration model was formulated based on the concept that the concentration of DnaA protein determines the initiation mass and the findings of titration of DnaA protein by plasmids carrying DnaA boxes. The model incorporated detailed knowledge about regulation of *dnaA* gene expression, the eclipse period and the role of Dam, and the initiation synchrony of multiple origins in fast growing cells. In these last sections we will review the newer findings about the role of the *datA* locus as well as the DARS1 and DARS2 loci and the interconversion of the two nucleotide forms of DnaA (Figure 13) and evaluate their respective contributions to initiation control before concluding remarks about revision of the model for control of initiation to include these aspects.

## DnaA-ATP hydrolysis—HDA and *datA*

Replicative inactivation of DnaA (RIDA) through hydrolysis of DnaA-ATP to DnaA-ADP was first discovered *in vitro* and shown to be dependent on replication and to require the “IdaB” (now Hda) protein and the β-clamp (DnaN; Katayama et al., 1998). A large increase in the DnaA-ATP form is found at high temperature in the *dnaN59*(Ts) and several other temperature sensitive replication mutants (Katayama et al., 1998; Kurokawa et al., 1999), showing that RIDA is also working *in vivo*. The *hda* gene was found in a search for clones that complemented (new) mutants defective in F plasmid maintenance at permissive temperature and were Ts for growth (Kato and Katayama, 2001). The gene was named *hda* (homologous to DnaA) and the Hda protein has extensive homology to domain III of DnaA. The Hda protein was shown to be identical to the IdaB protein through *in vitro* complementation, and an *hda* null mutant was shown to have a high percentage of DnaA-ATP (Kato and Katayama, 2001). In this study the *hda*(Ts) strain was also characterized *in vivo* and found to have increased *oriC* DNA per mass and increased *oriC*/*terC* indicating “abortive replication.”

Until recently, there has been some controversy regarding the essentiality of the *hda* gene. In the initial identification of *hda* (Kato and Katayama, 2001) it was found that the gene could not be deleted and only conditional mutants could be obtained. A little later isolation of an *hda* null mutant was reported (Camara et al., 2003). The *hda* null mutants are, however, slowly growing under standard conditions (aerobically on rich medium at 37°C) and genetically very unstable and quickly acquire suppressor mutations of many different types (Riber et al., 2006; Charbon et al., 2011). The growth defects are due to replication fork collapse caused by oxidative DNA damage so *hda* mutants have virtually no growth defects when grown anaerobically (Charbon et al., 2014) and also grow well in minimal media (Charbon et al., 2017). When grown under either of these permissive conditions the *hda* mutants still exhibit a decreased initiation mass (0.70–0.75) and moderate asynchrony.

**Figure 14:**
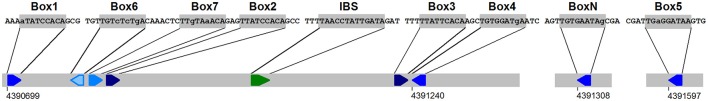
The *datA* region responsible for DDAH (*d**atA* dependent ATP hydrolysis, Kitagawa et al., 1998; Morigen et al., 2005). The DnaA boxes 2, 7, 3, and IBS are absolutely required for DDAH activity (Kasho et al., 2017). The coordinates are from *E. coli* K12 (acc.no. U00096.2). IBS: IHF binding site. The color code of the boxes is the same as in Figure 7. An additional one-misfit box (BoxN) is also indicated.

The *datA* locus was originally believed to exert its effect on regulation of initiation through titration of DnaA protein, similar to, but much stronger than, the titration by plasmids with the *mioC* or *dnaA* promoters (Kitagawa et al., 1998; Morigen et al., 2005). Later it was found that *datA* mediates DnaA-ATP hydrolysis of DNA bound protein, a process named DDAH (Kasho and Katayama, 2013). Only two of the five original DnaA-boxes (box 2 and 3) were absolutely required together with the IHF binding site (IBS) located between them. Further studies showed that a 2-misfit box next to box 2 (box 7, see Figure 14) was also essential for the DDAH activity (Kasho et al., 2017). The contribution of DDAH to DnaA-ATP hydrolysis is fairly small, in an otherwise wt strain deletion of *datA* increased DnaA-ATP a few % (Katayama et al., 2001) and when the *datA* deletion was introduced into the *rnhA* Δ*oriC*- Δ*hda* strain the percentage of DnaA-ATP was increased from 72 to ~90% (Kasho and Katayama, 2013).

Deletion of the *datA* locus has no effect on the growth rate of the bacteria but does affect the initiation control, leading to a decrease in the initiation mass (Kitagawa et al., 1998; Morigen et al., 2005; Frimodt-Møller et al., 2016). There is some controversy as to the degree of asynchrony conferred by deletion of *datA*. The first study which found a rather pronounced asynchrony in Δ*datA* cells (Kitagawa et al., 1998) also showed that such cells have close to the same DNA content and the same cell size distribution as wild type cells. Morigen et al. (2005) showed that much of the apparent asynchrony was due to rifampicin resistant initiations. It should be noted (emphasized) that rifampicin resistant initiations lead to overestimation of the effect on initiation mass. Rifampicin resistant initiation of chromosome replication has also been observed in *ihf* mutants, however, such initiations did not take place in strains carrying *ihf* and *dnaA46* mutations (von Freiesleben et al., 2000b) suggesting that the rifampicin resistant initiation phenotype of *ihf* mutants might be due to reduced function of *datA* in DDAH.

The phenotype of the Δ*datA* strain must primarily be due to the lack of DDAH activity since different point mutations inactivating the DDHA activity—mutation of the IBS, or inactivation of box 2 or box 7 have the same phenotype (Ogawa et al., 2002; Nozaki et al., 2009b; Kasho et al., 2017). The apparent very high titration activity of plasmids with the intact *datA* locus can largely be explained by the DDAH activity which lowers the DnaA-ATP level and gives increased expression from promoters which require this form of DnaA for repression. It is, however, clear that the *datA* locus also directly titrates DnaA protein by binding to the boxes, both from the initial deletion analysis of the *datA* plasmids where deletions outside the box 1–4 region gave reduced titration (Kitagawa et al., 1996) and from the analysis of plasmids with a box 2 or an IBS point mutation that only reduced titration (Nozaki et al., 2009b).

## DnaA-ADP rejuvenation

**Figure 15:**
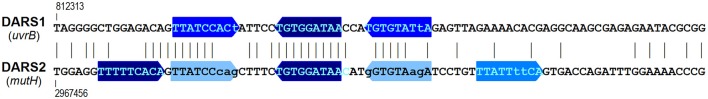
The DARSs (DnaA reactivating sequences). The coordinates given above and below the DARS sequences (Fujimitsu et al., 2009) are from *E. coli* K12 (acc.no. U00096.2). Vertical lines between the sequences indicate identical bases. See Figure 7 for color code for the boxes.

The first DARS DnaA-reactivating sequence, which is present in the ColE1 plasmid origin, was found somewhat fortuitously in a search for factors mediating *in vitro* DnaA-ADP to ATP exchange (Fujimitsu and Katayama, 2004). The full ColE1 DARS was delimited to a 70 bp sequence with three DnaA boxes, where two of the boxes in correct orientation were essential for activity. Subsequently two chromosomal DARSs were identified in the genome sequence amongst 52 pairs of similarly arranged DnaA boxes with 0 or 1 misfit to the sequence TTATNCACA (Fujimitsu et al., 2009). The DARS1 sequence is located in the *uvrB* promoter region and DARS2 in the *mutH* promoter (Figures 10, 15), both previously identified as sequences that interact with DnaA (van den Berg et al., 1985; Roth and Messer, 1998). The presence of extra DARS1 or DARS2 sequences on low copy number plasmids leads to decreased initiation mass (Fujimitsu et al., 2009; Charbon et al., 2011) and introduction of higher copy number plasmids with DARS1 or DARS2 is deleterious to the cells (van den Berg et al., 1985; Fujimitsu et al., 2009). Deletion of either DARS1 or DARS2 from the chromosome increases initiation mass by ~20% (Fujimitsu et al., 2009; Frimodt-Møller et al., 2016) and the two DARS sequences act synergistically since deletion of both increase initiation mass to close to 150% of the normal (Fujimitsu et al., 2009). Deletion of either DARS sequence reduce the DnaA-ATP percentage in the *rnhA* Δ*oriC*-Δ*hda* strain, and deletion of both DARS's almost returns the DnaA-ATP level to that of the *hda*^+^ strain (Fujimitsu et al., 2009). The effect of the DARS deletions, especially DARS2, is sufficient to give suppression of the lethality associated with lack of Hda mediated ATP hydrolysis (Fujimitsu et al., 2009; Charbon et al., 2011).

Intriguingly, it is only plasmids with DARS2, not with DARS1, that give asynchrony (Fujimitsu et al., 2009; Charbon et al., 2011), and only the DARS2 deletion that leads to asynchrony (Fujimitsu et al., 2009; Frimodt-Møller et al., 2016). This might be due to a higher efficiency of DARS2 in DnaA rejuvenation giving a stronger effect on DnaA-ATP % (Fujimitsu et al., 2009). It should be noted that all the data concerning initiation mass and asynchrony referred to above are from cells grown in minimal glucose casamino acids supplemented medium at 37°C. No systematic studies of the effect of DARS have been published for other growth media—i.e., slow growing cells.

We have summarized the effects of the different elements, de novo synthesis, RIDA, DDAH, and DARS on the interconversion of the two nucleotide bound forms of DnaA in Figure 13.

## Initiator titration revisited

A fundamental feature of the initiator titration model is that at the end of the eclipse (*oriC* sequestration) period the replication process has increased the number of DnaA boxes sufficiently to titrate so many DnaA proteins that there is not enough available for the low affinity binding at *oriC*. The additional feature with the two nucleotide forms of the DnaA protein and their interconversion does not essentially change this concept, in this case the amount of DnaA-ATP protein has to be reduced below a threshold during the eclipse period. Let us first consider a relatively slowly growing cell, e.g., 60 min doubling with a C time of 40 min. As soon as replication starts RIDA begins to work due to the presence of β-clamps remaining on the DNA after passage of the replisome, and when the *datA* locus is duplicated (8 min after initiation) the DDAH process will increase the hydrolysis rate a little. During these first approx. 10 min there is no DnaA protein synthesis and only one copy of the DARS sequences, and therefore the DnaA-ATP level will fall quickly. When the *dnaA* gene is no longer sequestered the DnaA-ATP dependent autorepression, will be low, and the DnaA-ATP level will start to increase due to synthesis of new proteins. At 16 min DARS2 is duplicated. In this relatively slow growing cell DARS2 might, however, be inefficient in nucleotide release since it requires Fis for this activity (Kasho et al., 2014) and Fis concentration is low in slow growing cells (Ball et al., 1992). At 26 min also DARS1 is duplicated and this will increase the building up of DnaA-ATP levels. At 40 min replication terminates and soon after RIDA will cease due to unloading of the β-clamps from the DNA which only takes a few minutes (Leu et al., 2000)^2^. In faster growing cells with overlapping replication cycles RIDA will be active throughout the cell cycle, but will vary with the number of replication forks, e.g., in a cell growing with 25 min doubling time the old forks will terminate 15 min after initiation, reducing the RIDA activity to two thirds, thus accelerating the buildup of DnaA-ATP. Unfortunately the variation in DnaA-ATP levels over the cell cycle has not been investigated using baby cells, it has only been measured in *dnaC* initiation synchronized cells (Kurokawa et al., 1999), which is an artificial situation probably with an unnaturally high DnaA-ATP starting level.

It will be interesting to incorporate the DnaA-ATP/ADP conversion systems into the initiator titration model and see how that behaves compared to the original initiator titration model, and to a version of the model where we insert the actual high affinity DnaA-box distribution.

It is clear that the original initiator titration model can to a large extent simulate the behavior of *E. coli* during exponential growth. And the *dnaA46* mutant with a protein that does not bind ATP (*in vitro*) is quite fit at low temperature, growing with an almost normal growth rate and having a reasonably uniform cell size distribution despite the asynchrony of initiation (and increased initiation mass). This raises the question why has *E. coli* (and other bacteria) evolved an additional level of regulation with the DnaA-ATP/DnaA-ADP interconversion systems? It will be interesting to see if these systems are necessary and under which growth conditions—how does an *E. coli* strain that is deleted for *hda, datA*, and the two DARSs behave? It will probably be necessary to simultaneously reduce the *dnaA* gene expression to get a cell with a normal initiation mass avoiding the side effects of over initiation.

## Author contributions

All authors listed have made a substantial, direct and intellectual contribution to the work, and approved it for publication.

### Conflict of interest statement

The authors declare that the research was conducted in the absence of any commercial or financial relationships that could be construed as a potential conflict of interest.

## Footnotes

^1^At the Cold Spring Harbor meeting in 1978 where the 422 bp sequence carrying *oriC* was presented (Messer et al., 1979), Hirota's group presented the complete sequence of a 2.1 kb BamHI fragment. After the meeting K. von Meyenburg and F. G. Hansen went to Arthur Kornberg's laboratory at Stanford University where we after comparing the sequences could see that the small 91 bp BamHI fragment was missing from the original presentation of the Japanese group. Therefore minichromosome pSY211, which was available at Stanford University, was restricted with *Bam*HI and analyzed on non-denaturing poly-acrylamide gels that allowed the separation of very small fragments. F.G.Hansen found that minichromosome pSY211 also contained 2 two small BamHI fragment. The sequence of the 91 bp BamHI fragments was added to the final report from the Japanese group (Hirota et al., 1979) and referred to as “Hausen and Schaller, personal communication.”

^2^The calculated average residence time for the β-clamps (3.5–7 min) is based on a very careful determination of the number of molecules of different polymerase subunits per cell, giving a number of 350 β-clamp dimers/cell in an exponentially growing culture of C600 in LB medium. However, the final calculation uses only 2–4,000 Okazaki fragments produced per cell in 40 min, corresponding to only one replicating chromosome per cell, but C600 growing in LB medium has mostly 8 origins per cell, and thus 4 pairs of replisomes, so the average residence time therefore has to be 3- to 4-fold shorter.
